# The multiple faces of self-assembled lipidic systems

**DOI:** 10.1186/1757-5036-2-3

**Published:** 2009-04-17

**Authors:** Guillaume Tresset

**Affiliations:** 1 Laboratoire de Physique des Solides, Université Paris-Sud, CNRS, UMR 8502, F-91405 Orsay Cedex, France

## Abstract

Lipids, the building blocks of cells, common to every living organisms, have the propensity to self-assemble into well-defined structures over short and long-range spatial scales. The driving forces have their roots mainly in the hydrophobic effect and electrostatic interactions. Membranes in lamellar phase are ubiquitous in cellular compartments and can phase-separate upon mixing lipids in different liquid-crystalline states. Hexagonal phases and especially cubic phases can be synthesized and observed *in vivo *as well. Membrane often closes up into a vesicle whose shape is determined by the interplay of curvature, area difference elasticity and line tension energies, and can adopt the form of a sphere, a tube, a prolate, a starfish and many more. Complexes made of lipids and polyelectrolytes or inorganic materials exhibit a rich diversity of structural morphologies due to additional interactions which become increasingly hard to track without the aid of suitable computer models. From the plasma membrane of archaebacteria to gene delivery, self-assembled lipidic systems have left their mark in cell biology and nanobiotechnology; however, the underlying physics is yet to be fully unraveled.

**PACS Codes:** 87.14.Cc, 82.70.Uv

## 1. Introduction

Lipids are the building blocks of cellular compartments. By self-assembling into bilayers, they form fluid membranes that act as relatively impermeable barriers to the passage of most water-soluble molecules. Lipid membranes enclose the cell machinery and protect it from the extracellular environment [1]. They likewise maintain the characteristic differences between the contents of each compartments and the cytosol. They accommodate a number of specialized molecules performing crucial functions to the life of the cell: ion channels pumping protons across the plasma membrane [2], nuclear pore complexes controlling access to and from the nucleus [3,4], or rotary motors synthesizing ATP [5,6]. Several of membrane proteins and glycosphingolipids are used as receptors by viruses and pathogens, including the Alzheimer's associated amyloid peptide [7-9].

Lipids share with other amphiphilic molecules the ability to self-assemble in solution into more or less complex aggregates, provided their density exceeds a certain critical micellization concentration (cmc) which depends upon their chemical structure and the ions present [10,11]. A typical cmc value for bilayer-forming lipids ranges from 10^-10 ^to 10^-6 ^M while micelle-forming lipids require 10^-5 ^up to 10^-2 ^M in the bulk solution [12]. The traditional view of the aggregation of amphiphilic molecules is based on the poor solubility of hydrocarbons in water, leading to what is known as the hydrophobic effect [13]. The presence of hydrocarbon residues induces the formation of a cavity in the water structure which causes an increased degree of order and consequently a significant decrease in the entropy of water [14,15]. When hydrocarbon residues meet upon an effective long-range attractive force [16,17], the cavities fuse with one another and expel water from the interface releasing entropy to the solution. This leads to the spontaneous formation of stable aggregates [18].

The hydrophilic headgroup – although not driving the aggregation – is responsible for the formation of an interface with water, and contributes to determine, in principle, the size and the shape of the aggregates through the interactions between the molecules. Simple geometric packing considerations allow the prediction of the final aggregate conformation given some elementary structural information on the amphiphilic molecules [19]. For this purpose, a geometric factor can be conveniently used, the dimensionless packing parameter *p*, defined as *p *≡ *v*/*a*_0_*l*_*c *_where *v *is the hydrocarbon volume, *a*_0 _the optimal headgroup area, and *l*_*c *_the critical chain length beyond which the hydrocarbon chain can no longer be considered as fluid [12]. This parameter determines whether the amphiphiles will form spherical micelles (*p *< 1/3), non-spherical micelles (1/3 <*p *< 1/2), vesicles or bilayers (1/2 <*p *< 1), or inverted structures (*p *> 1). This heuristic picture holds as long as only one amphiphilic component enters the system. Otherwise, the interactions between components – electrostatic interactions, van der Waals forces, or hydrogen bonding – may reorganize the system following a complex phase diagram. For example, the mixing, in the absence of added salt, of cationic and anionic surfactants with different packing parameters, yields a segregation of the amphiphiles and gives rise to unexpected aggregates such as nanodiscs, punctured planes, and facetted icosahedra, depending on stoichiometry [20-23].

Due to their natural occurrence in living organisms, lipids, and the assemblies they generate, are of special interest not only for the understanding of the many biological functions they are involved in, but also in regard of their applications as biocompatible carriers of drug and gene for pharmaceutical and biomedical purposes [24,25]. Another reason for this interest lies in a high potential in material science and nanobiotechnology, for example, by constructing intricate nanoscale networks of enzymatic reactors [26,27], or by arranging inorganic materials with the liquid-crystalline regularity of lipid complexes used as templates [28].

This article gives an overview of the various structures and arrangements based on lipids which have attracted the attention of biophysicists in the last few years. The functions of particular lipid structures within the cell are presented and the applications in therapeutic treatments or nanobiotechnology are mentioned whenever applicable. Emphasis is also given to the underlying physics that governs self-assembly processes and vesicle formation. The review begins with lamellar membranes along with a discussion on the phase separation occurring in raft microdomains. Afterwards, the varying forms of non-lamellar phases are described. The long-range (> 20 nm) organized structures come next, including liposomes, exotic vesicles and tubular objects. The last section encompasses complexed systems where lipids are associated with other entities, namely biological polyelectrolytes and inorganic materials. The review ends with a short section which highlights the benefits given by computer simulations in complementing experimental data to visualize and to understand the mesoscale structure of self-assembled lipidic systems.

## 2. Various aspects of lipid membranes

### 2.1 Lipid bilayer and lamellar phase

Bilayers are certainly the most common structure formed by lipids as they are present in every cellular organisms. They can take various shapes within the cell: fairly flat in the plasma membrane, spherical and tubular for the components involved in vesicular transport, or with an intricate geometry in the endoplasmic reticulum and Golgi apparatus. In this section, we focus on the short-range (over a few nanometers) organization of lipid bilayers.

**Figure 1:**
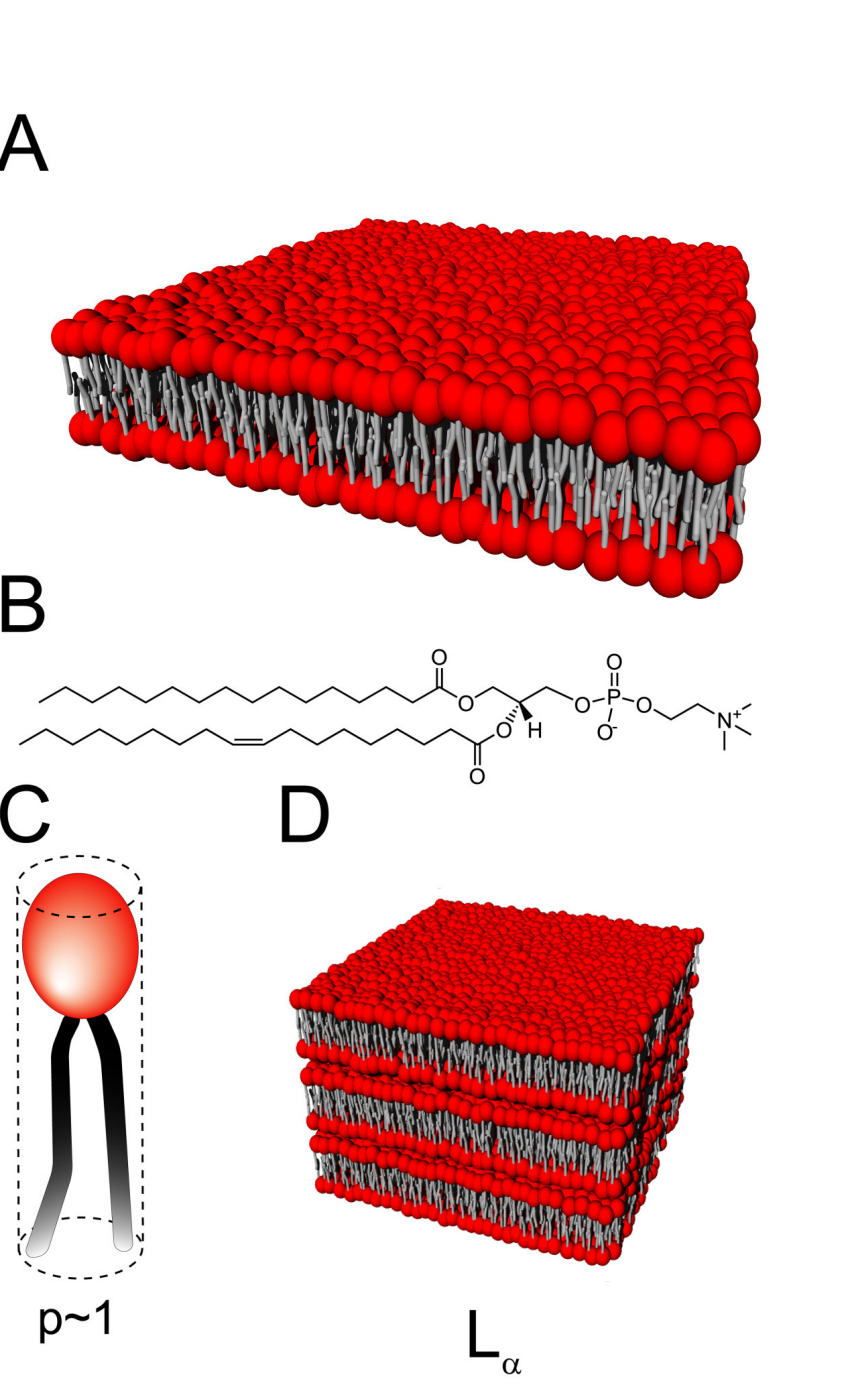
**Lipid bilayer**. (A) A flat membrane of lipids assembled into bilayer. (B) Chemical structure of 1-palmitoyl-2-oleoyl-*sn*-glycero-3-phosphocholine, a common phospholipid with *p *~1 and which thereby forms flat bilayers. A schematic lipid molecule is depicted in (C), with the hydrophilic headgroup represented in red and the double hydrophobic alkyl chain in grey. The molecule fits to a cylinder making its packing parameter *p *close to 1. (D) Lamellar phase *L*_*α *_of fluid lipid bilayers.

Figure 1A depicts a planar lipid membrane assembled into bilayer. This kind of flat membrane typically occurs for lipids with a packing parameter close to 1, which means that an individual lipid molecule fits to a cylinder (Figure 1C). Some of the phospholipids – one of the most frequently encountered family of lipids in nature [1,29] – have a tendency to form bilayer membrane, as is the case for 1-palmitoyl-2-oleoyl-*sn*-glycero-3-phosphocholine (POPC) shown in Figure 1B. The elastic properties of planar membranes are often described by the mean curvature modulus *κ *and the spontaneous curvature *c*_0 _[30,31]. The vast majority of bilayers in a biological context have an asymmetry – the interior and exterior of the cellular compartments – resulting in a finite spontaneous curvature. Yet recent studies by small-angle x-ray and neutron scattering showed that the inner and outer leaflets of vesicle bilayers can be indistinguishable, even for highly curved vesicles with diameters down to 62 nm [32]. The mean curvature modulus gives a measure of the membrane rigidity. Most biological membranes have *κ *≈ 30*k*_*B*_*T*, where *k*_*B *_is the Boltzmann constant and *T *the temperature, which makes them essentially flat at the molecular scale. *κ *depends upon the temperature and the bilayer composition – especially because of the interactions between the hydrocarbon chains of lipids [33,34] -, and contributes to the amplitude of membrane fluctuations [35].

Pure lipid bilayers are fluid at high temperatures but undergo a phase transition when the temperature decreases below a critical value [36]. The phase transition temperature is -2°C for POPC depicted in Figure 1B. According to its state, a lipid bilayer is said to be: in *L*_*α *_liquid-crystalline phase when it is fluid with melted hydrocarbon chains; in *L*_*β *_gel phase below the phase transition temperature; in  tilted phase when the gel phase tilts relative to the layer normal; and in  phase for tilted phase distorted by a periodic asymmetric ripple with a wavelength of the order of 100 Å [37,38]. The fluidity of lipid bilayer allows the membrane to reorganize spontaneously over a short time upon external stimulation: for instance, in response to an intense external electric field, biological membranes form submicrometer pores provided their transmembrane potential exceeds a critical breakdown value [39,40]. With no longer electrical stimulation, the pores reseal over a period ranging from milliseconds to a few seconds depending on the membrane dynamics. This technique, known as electroporation [41], is used to inject plasmid DNA across the plasma membrane of cells.

Notice that several bilayers can pile up with a thin layer of water solution separating each of them; such a structure is referred to as lamellar phase, denoted *L*_*α *_when the bilayers are fluid (Figure 1D). They are quite common with phosphatidylcholine (PC) lipids [36].

### 2.2 Phase separation and raft microdomains

A mixture of lipids in different phases – *L*_*α *_and *L*_*β*_, or liquid-disordered and liquid-ordered phases [42] for example – can phase-separate and give rise to the formation of raft microdomains in the bilayer. Each of the microdomains is enriched with lipids in the same liquid-crystalline phase. The size of microdomains ranges typically from a few nanometers to a few micrometers. Based on the properties of lipids in liposome membranes, domain models have long been proposed for native cell membranes [43,44]. The lateral segregation of lipids is believed to play a crucial role as it may govern a number of fundamental cellular processes such as signal transduction and inter and intracellular trafficking [45-48]. The self-organization into distinct domains permits the concentration of raft-associated specific receptors of proteins, which promotes the uptake of these proteins via the endocytic pathway. For example, a peptide sequence common to the Alzheimer's disease-associated A*β *peptide, the HIV-1 gp120 glycoprotein and the Prion protein was found to bind preferentially to raft-associated glycosphingolipids [7,49,50]. Such a peptide conjugated with a fluorophore constitutes a good raft marker for live cell imaging [51].

**Figure 2:**
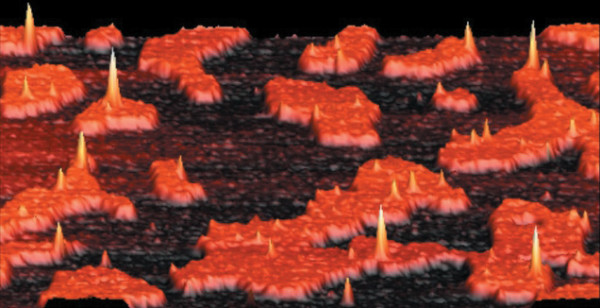
**Three-dimensional atomic force microscopy image of raft microdomains**. A binary mixture of 1,2-dioleoyl-*sn*-glycero-3-phosphocholine (black) and sphingomyelin (orange) forming a bilayer is immobilized on a mica substrate and exhibits a lipid phase separation. The height of the raft microdomains is ~7 Å. The yellow peaks correspond to a glycosylphosphatidylinositol-anchored protein which is located preferentially in the raft microdomains. The width of the scan is ~2 *μ*m. Adapted from reference [52]. Used with permission.

Biophysicists often investigate raft microdomains on supported lipid bilayers by atomic force microscopy (AFM), because this technique, unlike fluorescence microscopy, does not require the use of marker that may affect the phase separation of lipids [52-57]. Figure 2 shows a AFM image of a supported lipid bilayer on mica. The bilayer was made of a binary mixture of 1,2-dioleoyl-*sn*-glycero-3-phosphocholine (DOPC) in liquid-disordered phase and sphingomyelin in liquid-ordered phase. We can clearly see the domains of sphingomyelin emerging from the background of DOPC due to their larger size. A raft-associated protein is also visualized almost exclusively in the raft microdomains as expected [52].

Whether such idealized situations are transposable to live cells is still lively debated. Experiments on native lipid mixtures extracted from pulmonary membranes have shown the separation of two fluid phases [58], but the direct observation on live cell remains almost impossible due to the presence of anchored proteins and receptors that cover the membrane surface. Moreover, it seems that different experimental methods probe their own typical available length scales and therefore result in biased data. In an interesting computer study, Yethiraj and Weisshar [59] modeled a binary lipid mixture by using an Ising model on a square lattice comprising obstacles that mimic proteins anchored to the cytoskeleton. They reported that even at 5–10% by area of protein obstacles, the phase separation of lipids was dramatically reduced. This finding might bring the size of possible raft microdomains in live cell down to a few nanometers at best. However, another recent study reported that at physiological temperature, raft microdomains in the plasma membrane of an epidermoid carcinoma cell line coalesce upon the binding of cholera toxin B subunit to raft-associated ganglioside GM1, leading to the formation of raft clusters of a few micrometers in size [60].

### 2.3 Non-lamellar membrane structures

Lipids with packing parameter *p *~1 form preferentially bilayers, or more generally, a lamellar phase made of bilayer sheets. For other classes of lipids and mixtures of lipids, the three-dimensional polymorphism can be quite diverse, accompanied by a complex phase diagram depending on temperature, pressure, molecular structure and concentration of components [61]. The pioneering work in this field was carried out by Luzzati and coworkers who studied lipid-water systems by x-ray scattering techniques and found a number of non-lamellar liquid-crystalline arrangements which can be categorized into hexagonal and cubic phases [62-64].

**Figure 3:**
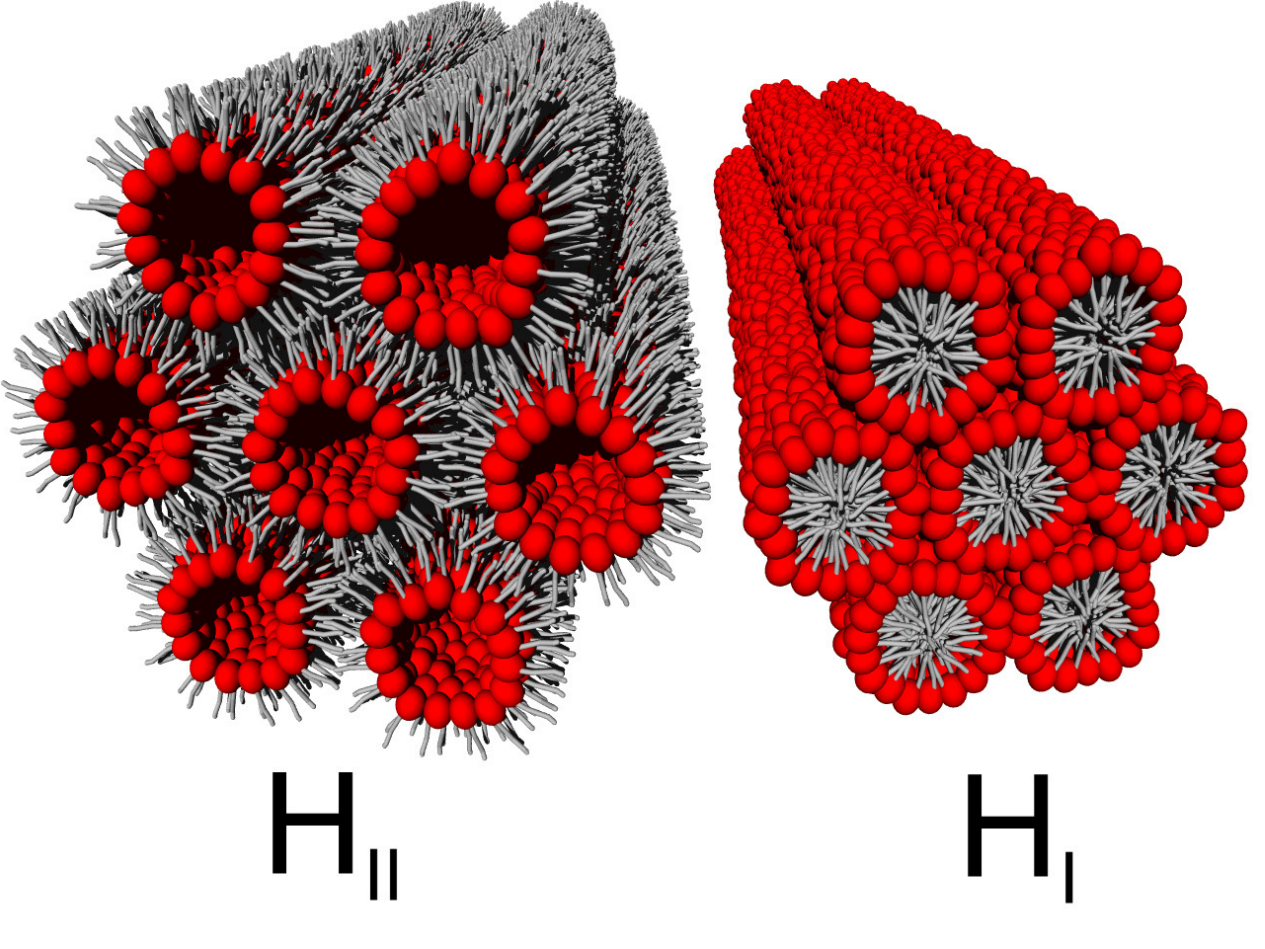
**Illustration of hexagonal lipid phases**. Inverted hexagonal (*H*_*II*_) and micellar hexagonal (*H*_*I*_) phases. The lipids are represented with the same conventions as on Figure 1, the hydrophilic headgroup in red and the hydrocarbon chains in grey.

Hexagonal phases are made of thin lipid cylinders with a radius of a few nanometers and arranged on a two-dimensional hexagonal lattice (Figure 3). When the polar headgroup of lipids is oriented towards the internal space of the cylinder, which is filled with water, the structure is called inverted hexagonal phase or *H*_*II *_[65]. In contrast, when the internal volume is filled with the hydrocarbon chains, the phase is said to be micellar hexagonal *H*_*I*_. Phosphatidylethanolamine (PE) is a class of lipids abundantly found in biological membranes and prone to form an inverted hexagonal phase [66]. At 20°C the radius of the water core in the cylinder is 15.9 Å for 1,2-dioleoyl-*sn*-glycero-3-phosphoethanolamine (DOPE) whose molecular length is 20.8 Å as inferred from x-ray diffraction reconstruction [67]. Below 20°C, DOPE can form a fluid lamellar *L*_*α *_phase in coexistence or not with an inverted hexagonal *H*_*II *_phase as the water content varies above ~10% (w/w) [68,69]. The propensity of PE lipids to form an inverted phase may be of high importance in relation to membrane fusion events. PE lipids may help their host membrane achieve highly curved intermediate structures during fusion, which is energetically favorable for the process [70,71].

**Figure 4:**
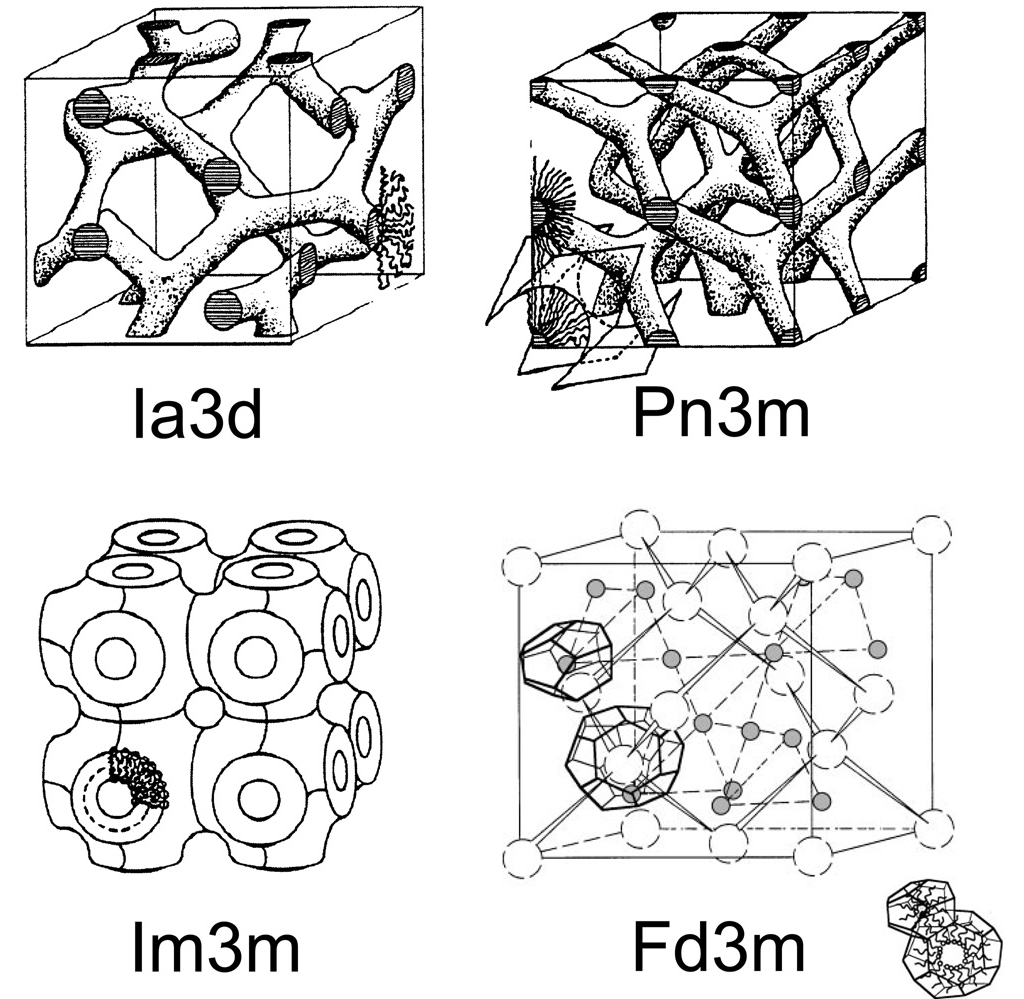
**Schematic structures of lipid cubic phases**. *Ia3d*, *Pn3m *and *Im3m *are the inverse bicontinuous cubic phases reported so far experimentally. *Fd3m *is an inverse micellar cubic phase found with mixtures of DOPC and glycerol. The two types of inverse micelle (open and grey spheres) with their polyhedral shapes are indicated on each site of the cubic lattice. Adapted from reference [73]. Reproduced by permission of the PCCP Owner Societies.

**Figure 5:**
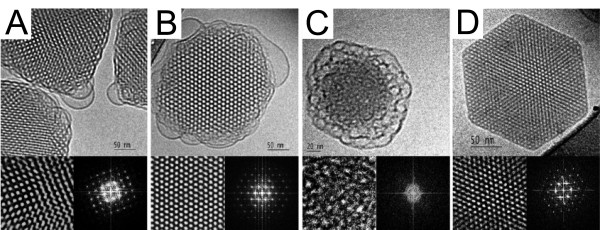
**Cryo-Transmission Electron Microscopy micrographs of non-lamellar lipid nanoparticles**. Inverse bicontinuous cubic phase nanoparticles viewed along the [001] (A) and [111] (B) directions. The Fourier transforms of magnified areas shown in the right-lower inserts of each micrographs are consistent with a body centered cubic phase *Im3m*. The nanoparticles are made up of a dispersion of glycerol monooleate (GMO), surfactants and polymeric stabilizers. (C) Sponge phase *L*_3 _nanoparticles containing diglycerol monooleate (DGMO) and glycerol dioleate (GDO). (D) Inverted hexagonal *H*_*II *_nanoparticles also based on DGMO and GDO but with a different fraction of stabilizer. Adapted from reference [75] with permission. Copyright 2005 by the American Chemical Society.

The other category of non-lamellar structures is made of three-dimensional cubic phases which are subdivided into bicontinuous and micellar classes [72,73]. The inverse bicontinuous cubic phases consist of a single continuous curved lipid bilayer folded into a three-dimensional cubic network and separating two disjointed water compartments. Following the mathematical argument of periodic minimal surfaces [73,74], the inverse bicontinuous phases can exhibit three distinct morphologies [75,76] labelled *Ia3d*, *Pn3m *and *Im3m *(Figure 4), the latter being not well established experimentally. The additional sponge phase (*L*_3_) can be viewed as a melted cubic phase because it shares the properties of bicontinuous cubic phases but does not have a long-range order [77]. In the micellar cubic phase, the structure is made up of disjointed inverted micelles packed on a cubic lattice. The micelles are actually distributed in two populations of different sizes to allow a more efficient packing of space. This is the case for phosphatidylcholine-glycerol mixtures which form a *Fd3m *micellar cubic phase [78]. Figure 5 provides a close-up view on the internal structure of lipid nanoparticles exhibiting bicontinuous cubic, sponge, and inverted hexagonal phases.

There are many evidences that lipid membranes in cubic phase are ubiquitous in the biological world. They have been observed in the plasma membrane of archaebacteria [79], as well as in the endoplasmic reticulum and mitochondria of mammalian cells [80]. In structural biology, lipid cubic phases can be employed as matrices to crystallize membrane proteins enabling diffraction and thereby reconstruction with a high resolution [81-83].

## 3. Vesicular and tubular shapes

### 3.1 Liposomes

**Figure 6:**
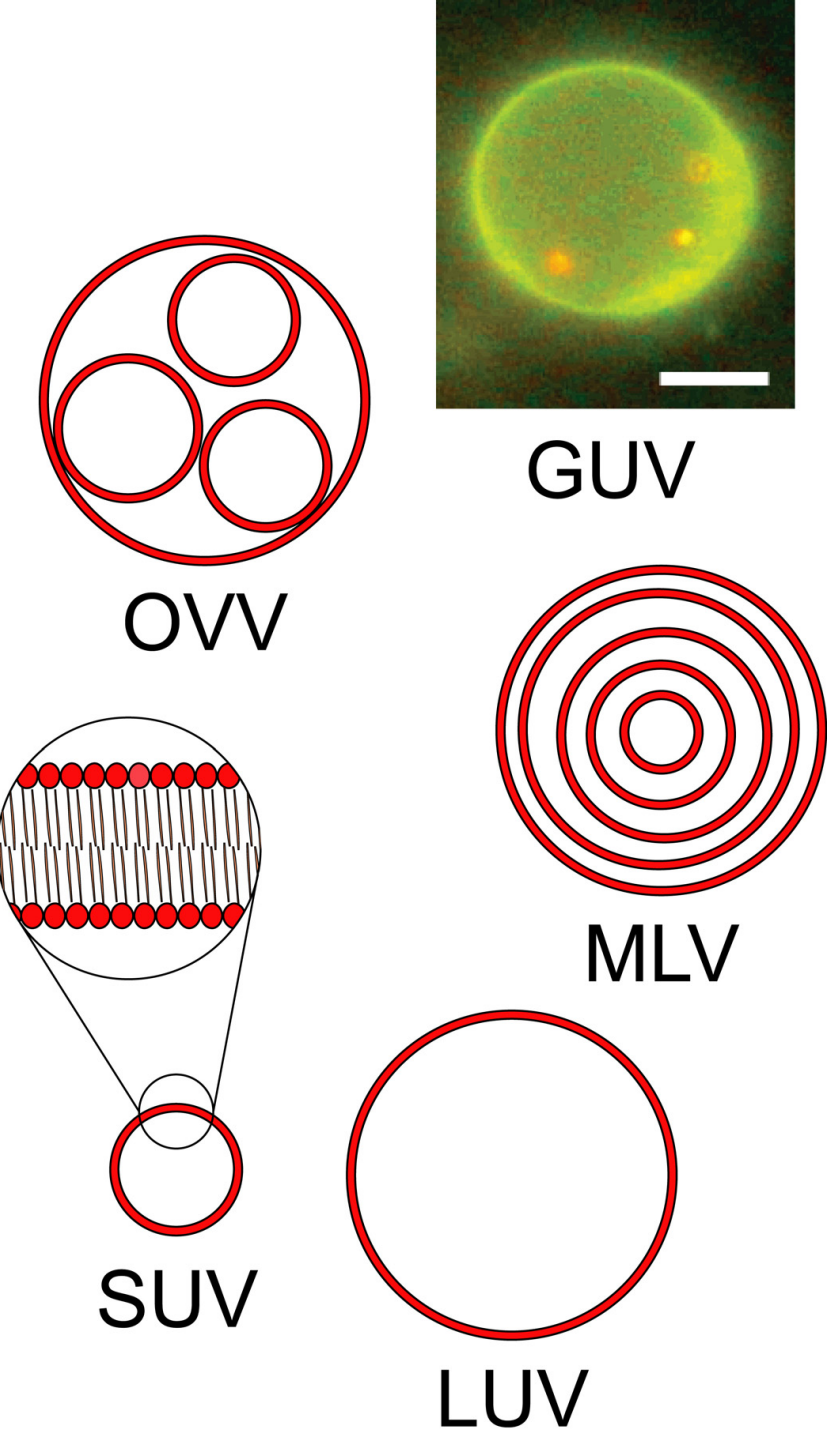
**Liposome morphologies**. Liposomes are presented with respect to the shape, size and number of bilayers. SUV = small unilamellar vesicle, LUV = large unilamellar vesicle, MLV = multilamellar vesicle, GUV = giant unilamellar vesicle, OVV = oligovesicular vesicle. The short-range structure of the lipid bilayers is shown in the magnified view. The GUV on the fluorescence picture is made of a mixture of phospholipids and fluorescent dye, and contains three red-fluorescent 200-nm polystyrene spheres which can move freely within the vesicle. The scale bar is 5 *μ*m. Adapted from reference [99] with permission. Copyright 2005 by the American Chemical Society.

When lipids are dispersed in an excess of aqueous solvent, they are no longer able to form a continuous phase and make instead a suspension of aggregates exhibiting locally one of the phases described earlier. For a lipid component with a packing parameter between 1/2 and 1, the resulting aggregates are spherical vesicles comprising one or several bilayers, and are called liposomes [25,84,85]. Liposomes come in varying size and lamellarity (Figure 6) [86]: the nomenclature usually distinguishes the small unilamellar vesicles (SUV, 10~100 nm), the large unilamellar vesicles (LUV, 100~1000 nm), the multilamellar vesicles (MLV, with an onion-like layered membrane), the oligovesicular vesicles (OVV, small vesicles incorporated into a bigger one), and the giant unilamellar vesicles (GUV, > 1 *μ*m), but other morphologies frequently occur as well. Liposomes can be prepared by spontaneous swelling of a lipidic film hydrated with an excess of the desired aqueous solution [87]. After formation, they are in general not colloidally stable and slowly aggregate and fuse into larger and more lamellar structures.

Submicrometer liposomes can be obtained with a narrow size distribution. Given that their membrane is biocompatible and impermeable to hydrophilic molecules, they can be conveniently used as nanocapsules. Consequently, submicrometer liposomes have attracted a strong interest in the biomedical and pharmaceutical sectors for their applications in drug delivery [24,88-90]. Liposomes are not just merely passive capsules transferring drugs into cells, their membrane can be engineered, for example so as to release the cargo inside a low pH environment such as in the endosome [91]. Many kinds of site-specific ligands such as antibodies, receptors and peptides can be anchored to the membrane, directing the cargo to designated cell types [92]. The grafting of poly(ethylene glycol) (PEG) at the surface of a liposome carrier enables an extended circulation lifetime in the body [93]. Other applications include the use of liposomes as marker for ultra sensitive detection of biological toxins [94,95], or in binding assays of peptides to membrane receptors [51,96].

Giant vesicles occupy a privileged place in biophysics because their micrometer size allows a direct observation under optical microscope. They can be conveniently used as model of cell membrane for investigating biological processes in controlled environment, as well as for bioanalytical purposes [27,97]. The electrically-induced fusion of giant vesicles gives insights into the response of biological membranes to electric fields; it reveals for instance the existence of a threshold intensity related to the critical transmembrane potential [39,98-101]. The activity of particular ion channels embedded into giant liposomes can be recorded via patch-clamp methods [102]. Giant vesicles also constitute a good biomimetic environment for monitoring enzymatic reactions [103,104], and more fundamentally, they can be envisioned as a minimal system for constructing an artificial cell assembly expressing genes [105-107].

### 3.2 Exotic vesicles

The shape of lipid vesicles at equilibrium is not limited to a sphere. A large variety of shape deformations on giant vesicles are achievable by changing the external constraints on the membrane, namely the osmotic pressure difference between the interior and exterior of the vesicle [108] and the temperature [109]. The equilibrium shape of the vesicle can be fully determined by the area difference elasticity (ADE) model [110,111], which implies an additional term to the curvature energy of the membrane. This energetic term arises from the deviation in the total area difference between the inner and outer leaflets. Minimizing the thus-obtained free energy leads to the final shape. Complex two-dimensional phase diagrams can be numerically calculated, and the morphology of vesicles is then obtained as a function of a dimensionless measure of the volume-to-area ratio and of the intrinsic area difference which indicates the preferred curvature of vesicles [111].

**Figure 7:**
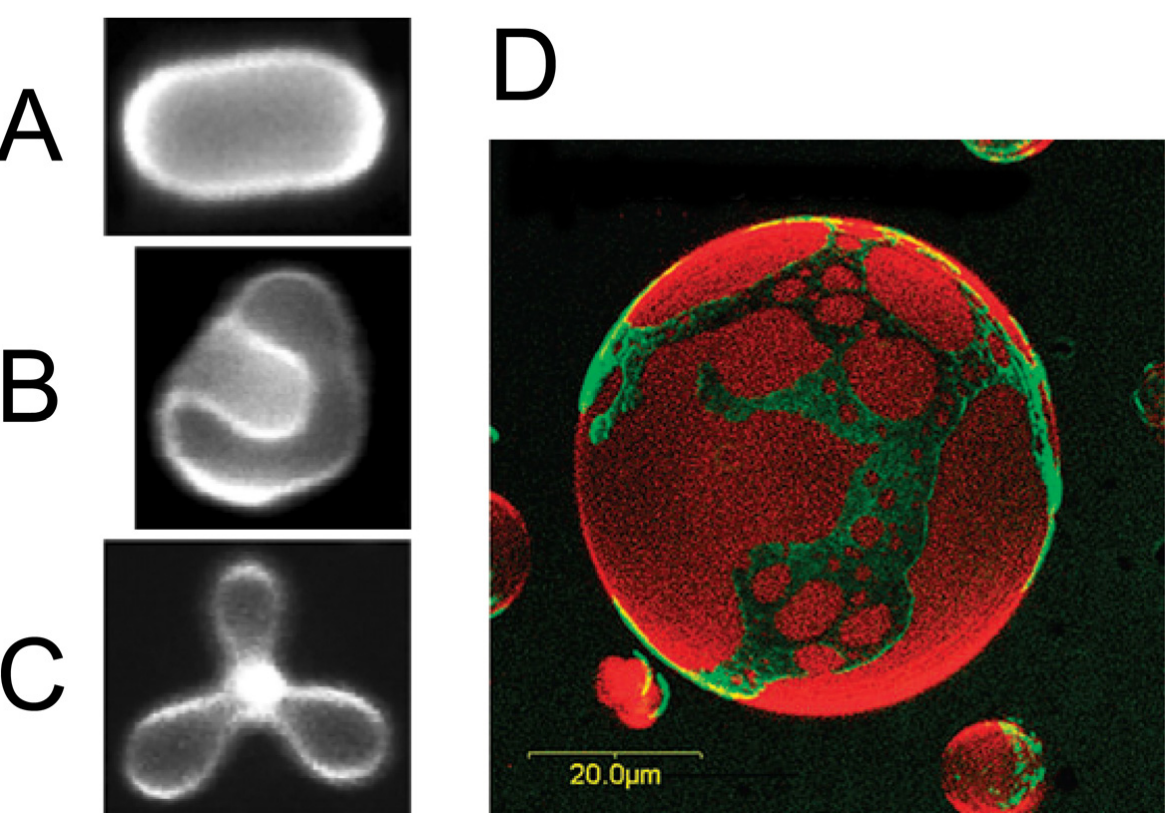
**Exotic vesicles**. (A) Prolate, (B) stomatocyte, and (C) starfish vesicles made of a ternary mixture of saturated and unsaturated phospholipids and cholesterol, in presence of ~1 mM of sorbitol at 60°C. Reprinted figure with permission from reference [114]. Copyright 2008 by the American Physical Society. DOI: 10.1103/PhysRevLett.100.148102 (D) Three-dimensional confocal microscopy image of a giant vesicle labeled with two distinct fluorescent dyes staining the liquid-ordered and liquid-disordered phases of a ternary mixture of lipids. Reproduced from reference [123] by permission of the PCCP Owner Societies.

Such calculations predict for phospholipid vesicles, a structural hierarchy of unexpected shapes such as rackets, boomerangs, and starfishes [112,113], and point out the existence of a critical point in the phase diagram where minute variations of membrane parameters can induce large shape transformations. Other shapes include stomatocytes, discocytes, prolates, and pears (Figures 7A, B and 7C) and have been reported experimentally with ternary mixtures of fluid lipids by adding salt into the extravesicular solution so as to apply an osmotic pressure difference [114].

In the case where the membrane of vesicles experiences a phase separation into raft microdomains, a term arising from the line tension at the phase boundary must be added to the free energy [115]. It results in complicated morphologies where the domains impose locally their preferred curvature and generate budding portions on the surface of vesicles [116-118]. Two photon fluorescence microscopy on giant vesicles provides a direct way to visualize lipid domains labeled with distinct fluorescent dyes. It gives information about the deformations induced on the vesicles (Figure 7D) [119-122] and allows to evaluate quantitatively the dynamics of raft microdomains [114,123,124].

### 3.3 Lipid nanotubes

Electron microscopy has revealed the existence of tubulo-vesicular elements interposed between the endoplasmic reticulum and the Golgi apparatus in pancreatic rat cells [125]. It was suggested that these lipid nanotubes, abundant around the Golgi complex, interconnect adjacent Golgi elements and are involved in the transport of membrane outward along microtubules [126]. The directed transport of small membrane blebs along a lipid nanotube has been observed in red blood cells as well [127], supporting the idea of the general interconnection of cellular compartments by lipid nanotubes.

Lipid nanotubes consist of multiple lipid bilayers rolled up in a long cylinder [128,129]. Their inner diameter ranges from ~10 nm with synthetic lipids to hundreds of nanometers for natural phospholipids, and their length can reach up to several centimeters [130].

Because most phospholipids do not self-assemble into tubular shapes upon simple dispersion, phospholipid nanotubes must be obtained for example by pulling on the membrane of immobilized giant vesicles with a micropipette [131,132]. In doing so, complex tubulo-vesicular networks in which the transport of specific molecules between compartments is assured by controlled diffusion can be constructed in view of bioanalytical applications [27,132,133]. In other protocols, the lipid tube growth is induced by a fluid flow guided in microfluidic channels [130,134], or by the binding of steptavidin to biotinylated membranes [135].

**Figure 8:**
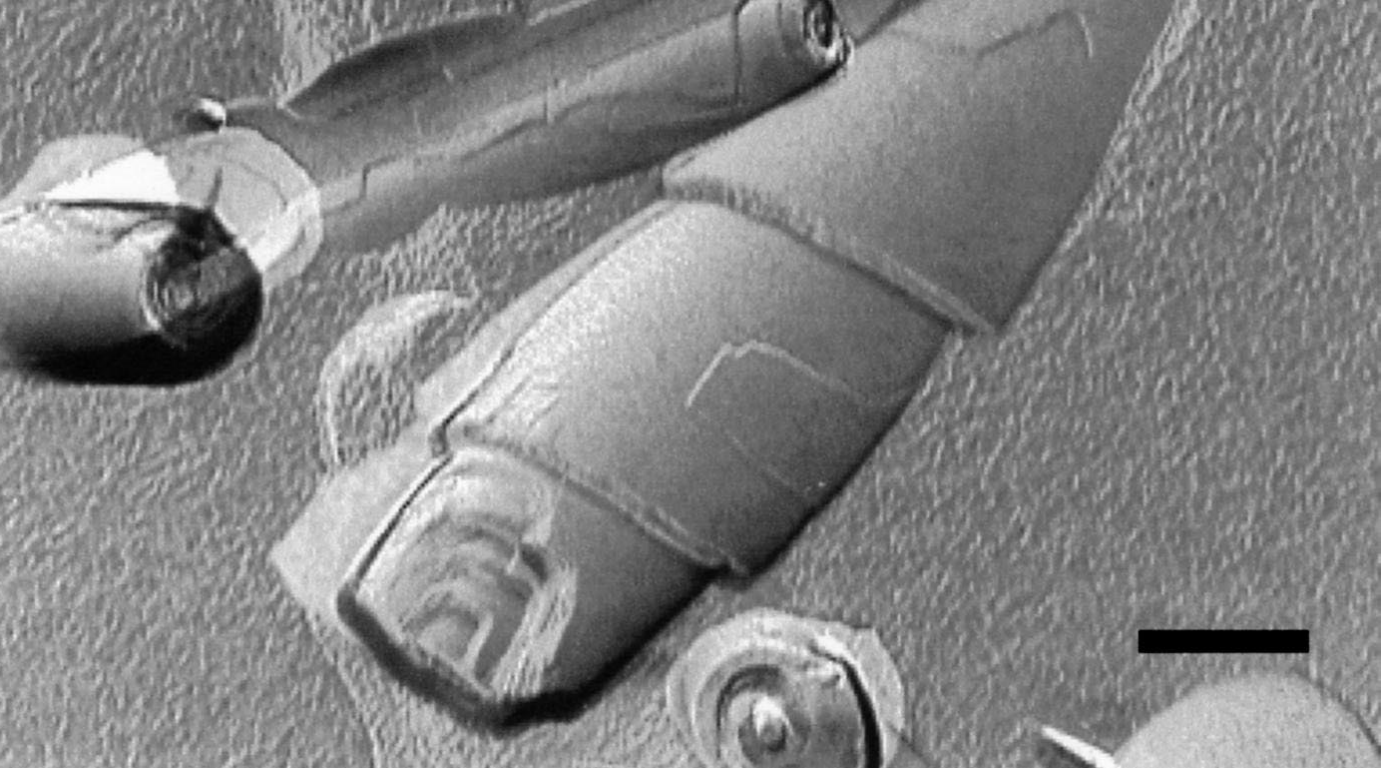
**Transmission electron micrograph of cochleates**. Transmission electron micrograph after freeze-fracture of cochleate cylinders prepared from anionic phosphatidylserine (PS) and calcium ions. The layered structure is clearly visible. The scale bar is 200 nm. Reproduced from reference [138] with permission. Copyright 2002 by Elsevier Science.

It is worth noting that synthetic polymerizable phospholipids have enabled the spontaneous assembly of nanotubes with diameters of approximately 500 nm upon cooling down below the gel-to-liquid crystalline phase transition temperature [136,137]. Besides, elongated assemblies called cochleates have been derived from dioleoylphosphatidylserine (DOPS), simply by adding calcium chloride to a dispersion of liposomes. Cochleates are made of spiral multilayered structures (Figure 8) held together by a cation bridge, and have been used in drug delivery [138].

**Figure 9:**
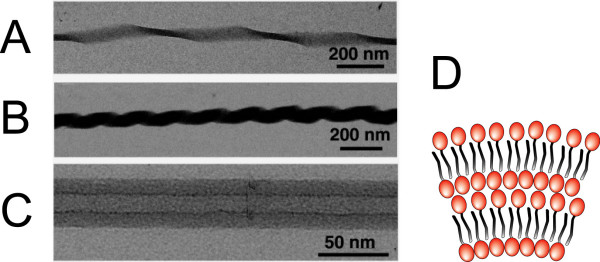
**Morphologies of glycolipid nanotubes**. Transmission electron microscope images of (A) twisted, (B) coiled, and (C) tubular one-dimensional assemblies of glycolipids. Reproduced from reference [128] with permission. Copyright 2005 by the American Chemical Society. (D) Schematic cross-section of the interdigitated lamellar layers of single hydrocarbon chain glycolipids in the nanotube membrane. The glycolipid headgroup is represented in red and the hydrocarbon chain in grey.

Following the spirit of research conducted on carbon nanotubes, studies on lipid nanotubes with attoliter void volume have been carried out. The typical nanotube diameter must lie in the range of ~10 nm, a curvature inaccessible to most of the lipid bilayers. Synthetic glycolipids presenting a single hydrocarbon chain are able to self-assemble into nanotubes of interdigitated lamellar layers stabilized thanks to a *π*-*π *stacking mechanism. Their inner diameter is about 10~15 nm, and their wall thickness up to ~15 nm [139] conferring a tubular persistence length of about 5 cm, that is, fifty fold as high as that of microtubules [140]. Interestingly, these glycolipids yield nanotubes with varying morphologies according to the degree of unsaturation of their hydrocarbon chain: twisted ribbon, coiled ribbon or nanotube without helical marking (Figure 9). The water confined within these nanotubes was shown to be highly structured with respect to the bulk [141].

## 4. Lipid-based complexes

Lipids can be complexed with virtually any materials provided that the electrostatic interactions are favorable. It is therefore impossible to review all the existing structures in a comprehensive manner. We will limit ourselves to the systems that have been extensively studied or that present a particular interest to biophysicists.

### 4.1 Lipid-DNA complexes or lipoplexes

Mesoscale assemblies made of lipids and DNA are perhaps the most documented self-assembled lipid-based complexes because they are a good case study of the intermolecular interactions between lipids and polyelectrolytes, and most importantly because they hold great promises for the future of gene therapy and protein delivery into cells [142-147]. Lipid-DNA complexes, also called lipoplexes, were first introduced some 20 years ago by mixing cationic liposomes with DNA, and allowed the effective transfer and expression of genes in cultured cells [148]. The encapsulation of DNA was by far more efficient than previous techniques involving liposomes because the cationic charge of the synthetic lipids enabled a 100-%-efficiency association with the negatively-charged DNA.

**Figure 10:**
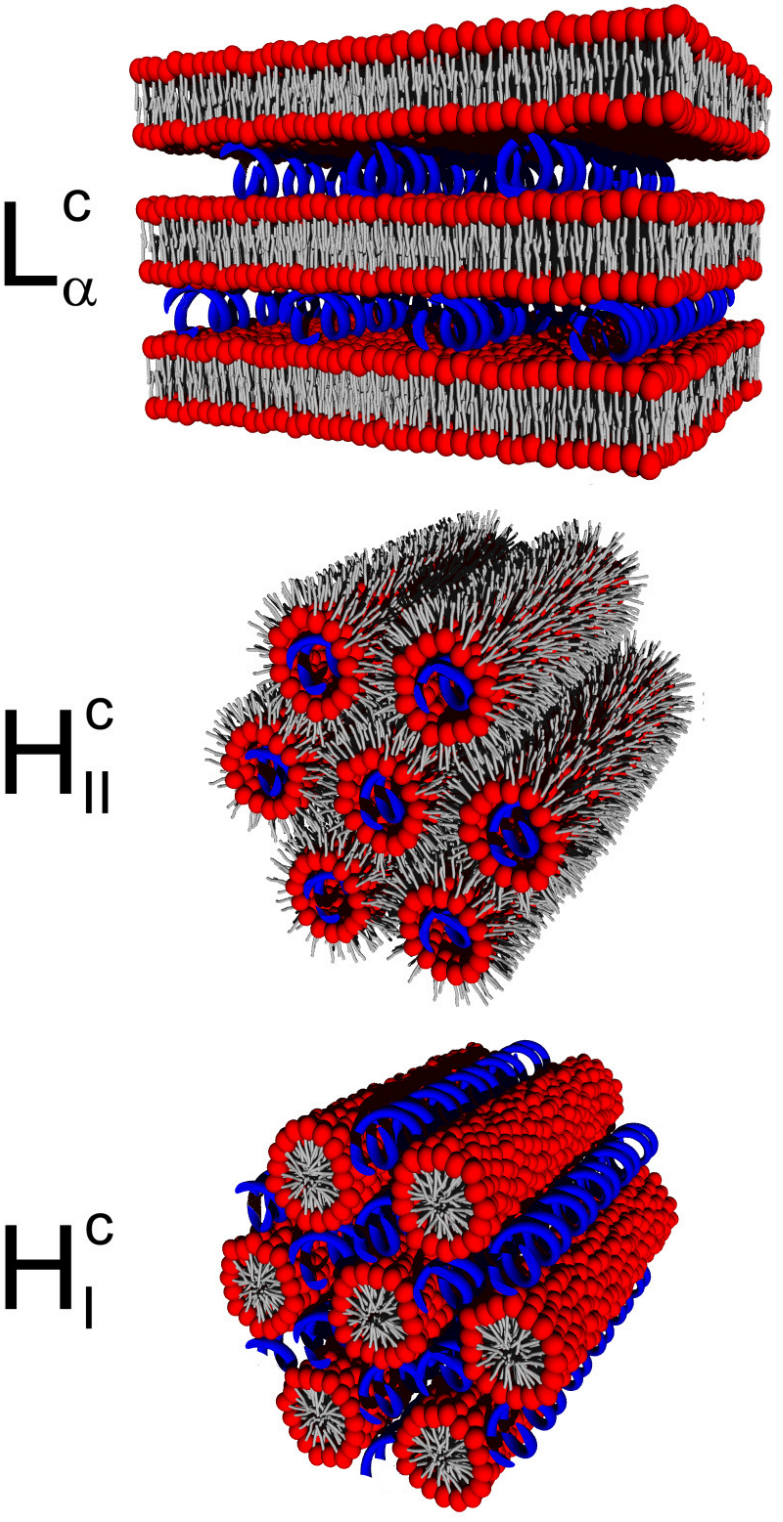
**Lipoplex phases**. Schematic representations of the phases of lipid-DNA complexes: complexed lamellar , complexed inverted hexagonal , and complexed micellar hexagonal . The lipids are depicted in red (headgroup) and grey (hydrocarbon chain), while the DNA rods are in blue.

Several of the liquid-crystalline structures listed previously are recovered by lipoplexes (Figure 10). The complexed lamellar phase  consists of alternating monolayers of parallel DNA rods and lipid bilayers [149-151]. The spacing between the two-dimensionally condensed DNA rods is extremely regular and varies from nearly close packing (~24 Å) to about 60 Å depending on the lipidic charge density [152] and on the ions that screen the electrostatic repulsion created by the rods [153]. Moreover, there is a transbilayer correlation in the DNA ordering possibly controlled by the membrane rigidity which varies with the temperature [154]. Another structure often encountered is the complexed inverted hexagonal phase  where DNA rods are coated by a lipid monolayer and arranged on a two-dimensional hexagonal lattice. In comparison with the lamellar phase , this structure is obtained either by increasing the spontaneous curvature of the membrane or by lowering its mean curvature modulus down to a few *k*_*B *_*T *[155]. A complexed micellar hexagonal phase  was reported as well, where the DNA rods are arranged on a honeycomb lattice in the interstices of the lipid micelle arrangement [156].

Cationic lipids scarcely occur in cell membrane, they are only found in tiny amounts in neuronal tissues as cationic glycosphingolipids for instance [157]. As a result, the injection of synthetic cationic lipids into cells induces a number of toxic effects, often lethal, the more so as the lipid-to-DNA charge ratio of lipoplexes increases [158]. To address this issue, non-cationic phospholipids have been used in association with multivalent cations. By binding to the lipid headgroup, multivalent cations are able to turn the overall headgroup charge positive [159], making the complexation with negatively-charged DNA electrostatically favorable. In doing so, the usual complexed liquid-crystalline structures are recovered, namely lamellar [160-162] and inverted hexagonal [163,164], the cations being intercalated so as to bridge the phospholipid headgroups and the DNA rods. Such systems have been proven as efficient as cationic lipids to transfer genes in cultured cells depending on the concentration and the valence of cations [164]. Monte Carlo calculations have shown that phospholipid-DNA complexes are the more stable in terms of free energy as the cation valence increases but this stabilization saturates beyond the value +4 [165].

The efficacy of gene transfection into cells depends upon a large number of variables and to date no clear picture has been drawn relatively to the requirements for an optimal delivery of genes. It is commonly admitted that lipoplexes are internalized by endocytosis after binding to the negatively-charged cell surface thanks to their cationic charge [166]. The charge may also play a role in promoting the fusion necessary to escape the endosome. However, at high lipid-to-DNA charge ratio, the DNA may be so strongly coupled to the lipids that it cannot be released toward the nucleus [167]. The liquid-crystalline structure appears to be critical for an efficient release of DNA. A good configuration seems to start from a stable lamellar  lipoplex, which turns into a non-lamellar – possibly non-complexed hexagonal or cubic phase – upon mixing with the anionic lipids of the endosomal membrane [168,169].

### 4.2 Other lipid-polyelectrolyte complexes

Mixtures of cationic and neutral lipids that yield membranes in lamellar phase have been used in association with negatively-charged filamentous bacteriophage M13 virus and cytoskeletal filamentous actin (F-actin). The two polyelectrolytes have similar diameters, ~6.5 nm for the former and 7.5 nm for the latter, but different surface charge densities, 1 e^-^/256 Å ^2 ^and 1 e^-^/625 Å ^2 ^respectively. Like DNA, M13 virus and lipids form a complexed lamellar phase  with an inter-M13 spacing of 8.2 nm, slightly larger than the diameter of the M13 virus [170]. In contrast, F-actin and lipids result in the formation of ribbon-like nanotube structures with an average width of 250 nm and length up to 100 *μ*m, consisting of lipid bilayers sandwiched between two layers of actin [171]. This difference of structure is attributed to the charge-density-matching mechanism: because the F-actin lattice of low charge density cannot compensate the charge density of the lipid membrane (1 e^+^/251 Å ^2^), the system self-assemble into a superlattice structure where one layer membrane is matched against two layers of F-actin.

Another unconventional complexed lamellar structure is produced with poly-L-glutamic acid (PGA) polypeptides. Small angle x-ray scattering data revealed a "pinched lamellar" structure where anionic PGA and cationic lipids formed localized pinched regions; in between, the adjacent quasi-neutral bilayers swelled into large pockets of water stabilized by hydration repulsion [172].

**Figure 11:**
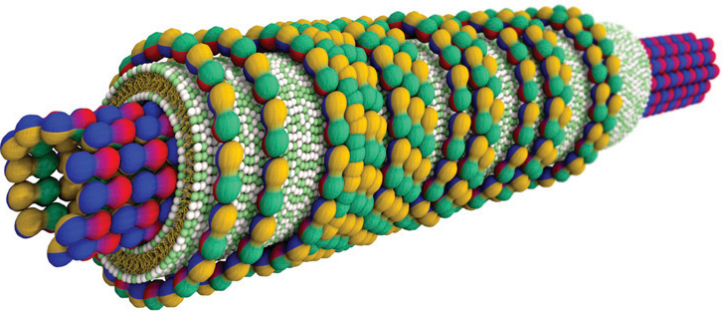
**Lipid-microtubule nanotube**. Complexed nanotube consisting of a microtubule (~26 nm in diameter) made of tubulin protein subunits (red-blue-yellow-green spheres) coated by a cationic lipid bilayer (headgroups in green and white, hydrocarbon tails in yellow). A third layer of tubulin oligomers actually wraps up the bilayer in the plane perpendicular to the nanotube axis. The arrangement is deduced from x-ray data and transmission electron microscopy images. Reproduced from reference [175] with permission. Copyright 2007 by the Biophysical Society.

The final structure of lipid-polyelectrolyte complexes arises from the interplay of the electrostatic interactions, the spontaneous curvature *c*_0 _and the mean curvature modulus *κ *of the lipid membrane, the polyelectrolyte itself being considered as rigid enough not to bend over length scale comparable to the liquid-crystalline periodicities. When the polyelectrolyte curvature is much higher than *c*_0_, the complex ends up in either  or  phase. If the curvatures fall into the same range as is the case with negatively-charged microtubules (~26 nm in diameter), the lipids form either a beads-on-a-rod structure along the polyelectrolyte at *κ *>> 10*k*_*B *_*T*, or at low *κ *(< 10*k*_*B *_*T*), they wrap it up in a bilayer to make a templated nanotube (Figure 11) [173-175].

### 4.3 Association with inorganic materials

Lipid bilayers immobilized on solid supports have become very popular for mimicking the basic processes occurring on a real cell membrane (see the section dedicated to raft microdomains) and for biotechnological applications [176,177]. A number of coupling techniques have been developed over the past decades including polymer-cushioned lipid bilayers [178,179], hybrid bilayers [180], tethered lipid bilayers [181] and physically self-assembled lipid monolayers [182], with the possibility to pattern the membranes on the micron scale by using photolithography [183]. The simplest route though is by the spreading of small lipid vesicles on hydrophilic substrates [184], employing if necessary divalent cations to bridge the like charges of lipids and substrate [185,186].

**Figure 12:**
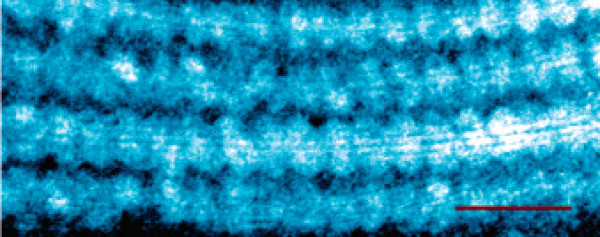
**Lipid bilayers templated on carbon nanotubes**. Transmission electron microscopy image of self-assembled single-chained lysophospholipids on single-walled carbon nanotubes. The assemblies display a striation periodicity of ~4.5 nm. The scale bar is 15 nm. Reproduced from reference [189] with permission. Copyright 2006 by the American Chemical Society.

In nanotechnology, the association of lipids with carbon nanotubes aims at functionalizing inorganic nanoobjects for bio-related applications. Carbon nanotubes are tubular assemblies of carbon atoms with inner diameters ranging from 1 to 10 nm and possess a number of attractive mechanical and electrical properties [187]. Intrinsically hydrophobic, they are poorly soluble in aqueous solution. Synthetic single-chain lipids designed for the immobilization of histidine-tagged proteins can successfully coat carbon nanotubes in monolayer [188]. Lysophospholipids, i.e. single-chained phospholipids, form striated arrangements with a ~4.5-nm periodicity [189] on the surface of single-walled carbon nanotubes (Figure 12), and improve dramatically their solubility [190]. Lipid bilayers can be also obtained around nanotubes by coating them with layers of oppositely charged flexible polyelectrolytes prior to liposome fusion [191]. These systems are anticipated to yield novel sensors, biosensors and photo-switched functional devices [192], and may be used for nanotoxicological studies [193].

## 5. Perspectives on computer simulations

Most of the studies described above relied on experimental methodologies to get structural information about the system of interest, often indirectly. Electron microscopy, x-ray scattering, atomic force microscopy, all these techniques give only certain features of the structure – symmetry or periodicity -, and must be supplemented with careful interpretations to reconstruct the detailed arrangement. The prediction of the final structure for a given system is challenging because a huge number of molecular interactions usually come into play. With the refinement of statistical mechanics models and the increasing rapidity of modern computers, fine structural calculations and dynamic over long time scale become accessible, for system complexity up to a limited extent though. We shall give a few words about the possibilities offered by computational techniques to self-assembled lipidic systems.

A lot of the underlying physics can be obtained by phenomenological Hamiltonians which conceive of the lipid systems as an assembly of thin interfaces characterized by their elastic constants. This description permits to deal with large systems, considering the collective behavior of lipid molecules possibly in interaction with polyelectrolytes. It is a very convenient approach to predict the equilibrium shape adopted by exotic vesicles [111]. We can also calculate the complete phase diagram of cationic lipid-DNA complexes as a function of the lipid composition and the lipid-to-DNA charge ratio [194,195]. This method, easy to implement numerically, yet requires an *a priori *knowledge of the system and of its behavior through the choice of suitable parameters. Density-Functional Theory (DFT) proceeds in a similar way, namely by assuming that the organized structures satisfy a local minimum of the free energy, this latter being represented in terms of molecular density-functionals [196,197]. Based on coarse-grained models of lipid molecules, DFT is able to reconstruct the phase diagram of lipid bilayers, predicting the transition from dilute bilayers to lamellar phase [198]. Applied to self-assembled systems, DFT is still in its infancy but holds many promises as it provides a rather fine structural description at a low computational cost.

**Figure 13:**
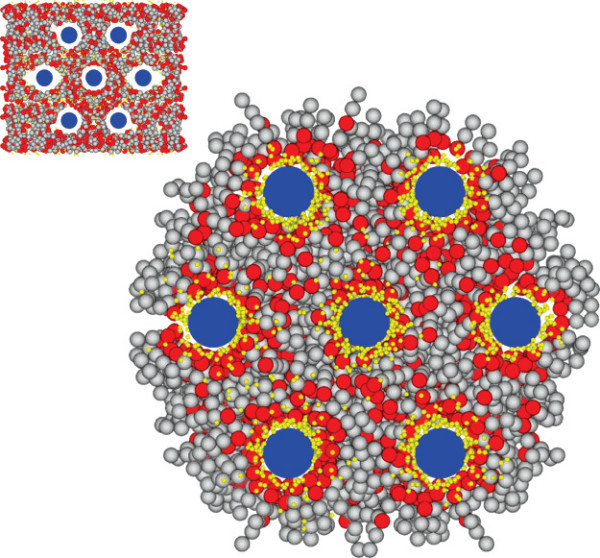
**Computer simulation of a self-assembled lipidic complex**. This water-free Monte Carlo simulation represents a hexagonal complex of zwitterionic lipids (headgroups in red and hydrocarbon chains in grey), divalent cations (yellow spheres) and DNA rods (blue). The DNA rods are maintained fixed on a hexagonal lattice over the simulation. Lipids and cations are randomly distributed at the initial stage (inset). Reproduced from reference [165] with permission. Copyright 2007 by the American Chemical Society.

A step further toward the real life is achieved by coarse-grained models implemented in Monte Carlo or molecular dynamic computer schemes [199,200]. In these models, group of atoms are lumped into pseudo-particles interacting via pair potentials. Noticeably, models with implicit solvent, in which hydrophilic/hydrophobic interactions are heuristically embedded into the pair potentials without the mediation of solvent molecules, enable to simulate large self-assembled lipidic systems with a decent accuracy and reasonable computational cost. They reproduce the elastic properties of weakly undulating lipid bilayers [201], as well as the self-assembly of lipid-DNA complexes [152,165,202]. Figure 13 shows a self-assembled lipid-ion-DNA complex in  phase simulated through a Monte Carlo scheme. Such a simple simulation gives quantitatively access to the thermodynamical stability of complexes in function of the valence of cations for instance. Other models, with explicit solvent, are able to reproduce the thermotropic lamellar-to-hexagonal phase transition of unsaturated phospholipids [203] or the two-dimensional phase separation occurring on the surface of binary fluid vesicles [204].

The ultimate refinement in molecular simulation is achieved by atomistic models in which the molecular structure and the interactions of components are described faithfully, including chemical bonds (bond, angle and dihedral potentials), electrostatic and van der Waals interactions [205]. Extremely accurate, they cannot, however, deal comfortably with mesoscale systems (extending beyond 10 nm) or track most of the self-assembly processes taking place beyond microseconds given the insufficient power of nowadays computers. Atomistic models have been up to now well suited for investigating the atomic interactions between lipids and proteins [206], lipids and DNA [207], or the configuration of lipids sticking around carbon nanotube [189], from a pre-assembled system close to its equilibrium, but they might reveal themselves as the method of choice for unraveling the full dynamic of self-assembly processes at atomic scale as soon as the computer technology will allow it.

## 6. Conclusion

**Figure 14:**
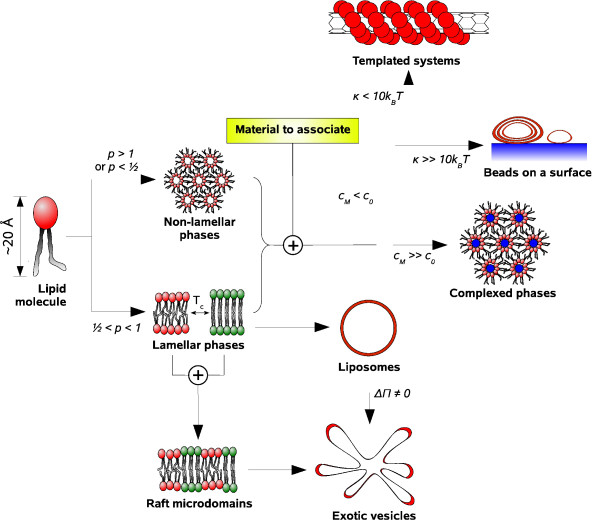
**Overview of the structures formed by self-assembled lipidic systems**. The structures described in this mini-review are summarized along with the physical factors enabling to get from one structural category to another. *p *denotes the lipid packing parameter, *c*_0 _and *κ *are the spontaneous curvature and the mean curvature modulus of membranes, *T*_*c *_is the critical temperature of lipid phase transition, ΔΠ is the difference of osmotic pressure between the interior of vesicles and the bulk solution, and *C*_*M *_stands for the local curvature of a material to associate. Each structural category is illustrated by a schematic of a typical lipidic structure: inverted hexagonal phase *H*_*II*_, fluid and gel lamellar phases *L*_*α *_and *L*_*β*_, raft microdomains, liposomes and starfish vesicles, complexed inverted hexagonal phase , lipid vesicles on a surface, and lipid-coated carbon nanotubes.

This short review has shown the diversity, the complexity and the multiscale nature of lipidic systems, no matter if they are purely made of lipids or in association with polyelectrolytes and inorganic materials. Figure 14 summarizes the various lipidic structures described before. When the packing parameter of lipids *p *is larger than 1 or smaller than 0.5, the system tends to form non-lamellar phases such as hexagonal and cubic phases. Otherwise (1/2 <*p *< 1), lamellar phases are obtained, with lipids in fluid or gel state depending on their phase transition temperature. Upon mixing lipids in different state, segregation into raft microdomains occurs. At the macroscopic scale, lamellar membranes form spontaneously spherical vesicles, or exhibit other shapes (tube, starfish, prolate etc.) when submitted to a constraint such as a difference of osmotic pressure between the interior of the vesicle and the bulk solution. In the presence of another material presenting a favorable electrostatic interaction with the lipids, three other structures are achieved depending on *c*_0_, the local curvature of the material *C*_*M*_, and the mean curvature modulus of lipid membrane *κ*. At high material curvature *C*_*M *_>> *C*_0_, complexed phases are generally obtained (,  etc.). If now the membrane curvature is higher than that of the material, either lipid vesicles with a stiff membrane (*κ *>> 10*k*_*B *_*T*) are immobilized on the material surface, or the lipid membrane is flexible enough (*κ *> 10*k*_*B *_*T*) to coat the material and results in a templated system.

If Biology finds naturally more interest in active compounds, that is, proteins and enzymes with dynamic and vital functions to the cell, Physics still remains intrigued and inquiring about the fundamental principles that drive the lipid self-organization into mesoscale structures covering three orders of magnitude in the nanometer range. Not only lipids may tell us about the spark that gave birth to primitive living organisms – Life is after all, a self-assembly process -, but also, by harnessing the building blocks of Life, we may be able to mimic, or even trick, Nature, and design Life-like systems performing specific tasks in a better way. No matter what the applications may be, protein crystallization or gene delivery, it is a safe bet that understanding self-assembled lipidic systems will continue to enrich the biological and medical research.
